# *De novo* transcriptome resources of the lichens, *Dirinaria* sp. UKM-J1 and UKM-K1 collected from Jerantut and Klang, Malaysia

**DOI:** 10.1016/j.dib.2018.07.020

**Published:** 2018-07-10

**Authors:** Izwan Bharudin, Siti NurhaniAbdul Abdul Rahim, Mohd FaizalAbu Abu Bakar, Siti Norsaidah Ibrahim, Shazilah Kamaruddin, Mohd Talib Latif, Mohd Wahid Samsudin, Abdul Munir Abdul Murad, Farah DibaAbu Abu Bakar

**Affiliations:** a School of Biosciences and Biotechnology, Faculty of Science and Technology, Universiti Kebangsaan Malaysia, 43600 Bangi, Selangor, Malaysia; b Malaysia Genome Institute, Ministry of Science, Technology and Innovation, Jalan Bangi, 43000 Kajang, Selangor, Malaysia; c School of Environmental and Natural Resource Sciences, Faculty of Science and Technology, Universiti Kebangsaan Malaysia, 43600 Bangi, Selangor, Malaysia; d School of Chemical Sciences & Food Technology, Faculty of Science and Technology, Universiti Kebangsaan Malaysia, 43600 Bangi, Selangor, Malaysia

**Keywords:** *De novo* assembly, RNA-seq, *Dirinaria* sp., Air pollution, Symbiosis, Gene discovery

## Abstract

Lichen is a symbiotic organism that exists as a single composite body consisting of a mycobiont (fungus) and a photobiont (algae or a cyanobacterium). Many lichen species are considered as extremophiles due to their tolerance to radiation, desiccation, temperature and pollution. However, not all lichen species are tolerant to harsh environmental conditions as several species are sensitive for example to nitrogen, sulphur, acidity, heavy metals, halogens (*e.g.* fluoride) and ozone. Thus, to better understand why some lichens can withstand exposure to pollutants as opposed to those that are susceptible, we focused on the lichen species of *Dirinaria* known for their wide distribution in the tropics, subtropics and pantropical, and moderate tolerance to air pollution. Their moderate tolerance to air pollution affords them to thrive in good air quality environments as well as polluted air environments. Lichen samples of *Dirinaria* sp., UKM-J1 and UKM-K1, were respectively collected from two areas with different levels of air quality based on Air Pollutant Index or API (with index pollutant criteria of PM_10_, carbon monoxide, ozone, nitrogen dioxide and sulfur dioxide) in the outskirt of Jerantut (UKM-J1), a rural area in the middle of Peninsular Malaysia and the township of Klang (UKM-K1), in a busy area of the Klang Valley, Malaysia. API was monitored throughout 2012–2013 whereby the sample collection site in Klang showed markedly higher concentrations of pollutants in all the index pollutant criteria as compared to that of Jerantut. We performed transcriptome sequencing using Illumina RNA-seq technology and *de novo* assembly of the transcripts from the lichen samples. Raw reads from both libraries were deposited in the NCBI database with the accession number SRP138994.

## Specifications Table

**Table:** 

Subject area	*Biology*
More specific subject area	*Environmental microbiology*
Type of data	*Transcriptome data*
How data was acquired	*Paired-end transcriptome of Dirinaria sp. was sequenced using Illumina HiSeq. 2000 at Malaysia Genome Institute. De novo transcriptome assembly was performed using Trinity RNA-Seq v2.4.0*
Data format	*Raw sequences (FASTQ)*
Experimental factors	*Samples were collected from two different sites with different air qualities based on Air Pollution Index (polluted air quality and good air quality)*
Experimental features	*Lichen Dirinaria sp. samples were collected from tree barks in Klang and Jerantut, Malaysia.*
Data source location	*Jerantut and Klang, Malaysia*
Data accessibility	*Raw FASTQ files were deposited in NCBI SRA database with the accession number*SRP138994 (https://www.ncbi.nlm.nih.gov/sra/?term=SRP138994)

## Value of the data

• The data obtained using Illumina sequencer is the first source of *Dirinaria* sp. RNA-seq.
• These data provide a glimpse into the molecular nature of the lichen *Dirinaria* sp. and contribute further to the understanding of lichen symbiosis.
• The data presented here can be used for gene discovery in examining the tolerance of this lichen towards air pollution.
• The data can also be used in unravelling genes and pathways involved in the synthesis of unique secondary metabolites.

## 1 Data

Two lichen samples collected from Jerantut (good air quality), Pahang and Klang (polluted air quality), Selangor, Malaysia (Table 1), were identified as *Dirinaria s*p. UKM-J1 and K1, respectively, through Polymerase Chain Reaction (PCR) and rRNA Internal Transcribed Spacer (ITS) sequencing [1]. Transcriptome data were generated from the total RNA extracted from these two lichen samples. Details of the experimental procedure and sequence analyses are described in the next section.

**Table 1:** Comparison of the average concentrations of five air pollutants throughout 2012–2013 between sample collection sites of Jerantut and Klang, Malaysia.

	Jerantut	Klang
Carbon monoxide, CO (ppm)	0.12	0.61
Sulphur dioxide, SO_2_ (ppb)	0.70	4.72
Nitrogen dioxide, NO_2_ (ppb)	2.21	21.10
Ozone, O_3_ (ppb)	22.61	28.81
Suspended particulates, PM_10_ (µg/m^3^)	33.94	62.87

## 2 Experimental design, materials and methods

### 2.1 Sample preparation

Lichen sample collection, RNA extraction procedure and library construction for data production were as previously described [1].

### 2.2 Assembly and RNA-seq analysis

The raw RNA-seq data from *Dirinaria* sp. were trimmed and filtered with SolexaQA++ [2] to acquire high-quality reads. Phred quality value of Q20 and reads longer than 50 bp were used as parameters. Paired-end reads were determined using Perl script *select_paired.pl* [3]. *De novo* assembly of high-quality reads was carried out using Trinity RNA-Seq v2.4.0 [4], with default parameters. Table 2 shows the RNA-seq statistics whereas the assembly statistics are as shown as in Table 3.

**Table 2:** Statistics of the RNA-seq generated from two different libraries.

	Jerantut	Klang
Raw reads	61,101,766	46,951,030
Clean reads	51,051,344	39,379,978
Read counts for transcriptome assembly (paired-end reads)	46,457,806	36,013,638
Average read length (bp)	89	90
Total base pair (bp)	4,125,748,902	3,233,294,069

**Table 3:** Assembly statistics using Trinity RNA-Seq v2.4.0.

Attributes	Value
Number of transcripts	379,310
Total residues (bp)	353,841,575
Average length (bp)	935
N50 transcript	1902
Largest transcript (bp)	14,535
Smallest transcript (bp)	201
